# Traditional Chinese medicine and new concepts of predictive, preventive and personalized medicine in diagnosis and treatment of suboptimal health

**DOI:** 10.1186/1878-5085-5-4

**Published:** 2014-02-13

**Authors:** Wei Wang, Alyce Russell, Yuxiang Yan

**Affiliations:** 1 School of Medical Sciences, Edith Cowan University, Perth, Western Australia WA6027, Australia; 2 Municipal Key Laboratory of Clinical Epidemiology, School of Public Health, Capital Medical University, Beijing 100069, China

**Keywords:** Suboptimal health, Suboptimal health status questionnaire-25, Chronic disease, Predictive, Preventive and personalized medicine

## Abstract

**Background:**

The premise of disease-related phenotypes is the definition of the counterpart normality in medical sciences. Contrary to clinical practices that can be carefully planned according to clinical needs, heterogeneity and uncontrollability is the essence of humans in carrying out health studies. Full characterization of consistent phenotypes that define the general population is the basis to individual difference normalization in personalized medicine. Self-claimed normal status may not represent health because asymptomatic subjects may carry chronic diseases at their early stage, such as cancer, diabetes mellitus and atherosclerosis. Currently, treatments for non-communicable chronic diseases (NCD) are implemented after disease onset, which is a very much delayed approach from the perspective of predictive, preventive and personalized medicine (PPPM). A NCD pandemic will develop and be accompanied by increased global economic burden for healthcare systems throughout both developed and developing countries. This paper examples the characterization of the suboptimal health status (SHS) which represents a new PPPM challenge in a population with ambiguous health complaints such as general weakness, unexplained medical syndrome (UMS), chronic fatigue syndrome (CFS), myalgic encephalomyelitis (ME), post-viral fatigue syndrome (PVFS) and chronic fatigue immune dysfunction syndrome (CFIDS).

**Methods:**

We applied clinical informatic approaches and developed a questionnaire—suboptimal health status questionnaire-25 (SHSQ-25) for measuring SHS. The validity and reliability of this approach were evaluated in a small pilot study and then in a cross-sectional study of 3,405 participants in China.

**Results:**

We found a correlation between SHS and systolic blood pressure, diastolic blood pressure, plasma glucose, total cholesterol and high-density lipoprotein (HDL) cholesterol among men, and a correlation between SHS and systolic blood pressure, diastolic blood pressure, total cholesterol, triglycerides and HDL cholesterol among women.

**Conclusions:**

The SHSQ-25 is a self-rated questionnaire of perceived health complaints, which can be used as a new instrument for PPPM. An ongoing longitudinal SHS cohort survey (China Sub-optimal Health Cohort Study, COACS) consisting of 50,000 participants will provide a powerful health trial to use SHSQ-25 for its application to PPPM through patient stratification and therapy monitoring using innovative technologies of predictive diagnostics and prognosis: an effort of paradigm shift from reactive to predictive medicine.

## Overview

Health is the level of functional or metabolic efficiency of a living life. In human beings, it indicates the general condition of a people's mind, body and spirit, usually meaning to be free from illness, stress, injury or pain [1,2]. The World Health Organization (WHO) defines health as ‘a state of complete physical, mental, and social well-being and not merely the absence of disease or infirmity’ [3,4]. Although the WHO definition of health has been subjected to controversy, in particular as having a lack of operational value and the problem created by use of the word ‘complete’, it remains the most enduring since it was promulgated in 1946 [5,6]. Like the International Classification of Diseases (ICD) for disease diagnoses, classification systems such as the Family of International Classifications, including the International Classification of Functioning and Disability and Health (ICF), are commonly used to define and measure the components of health [2].

**Figure 1:**
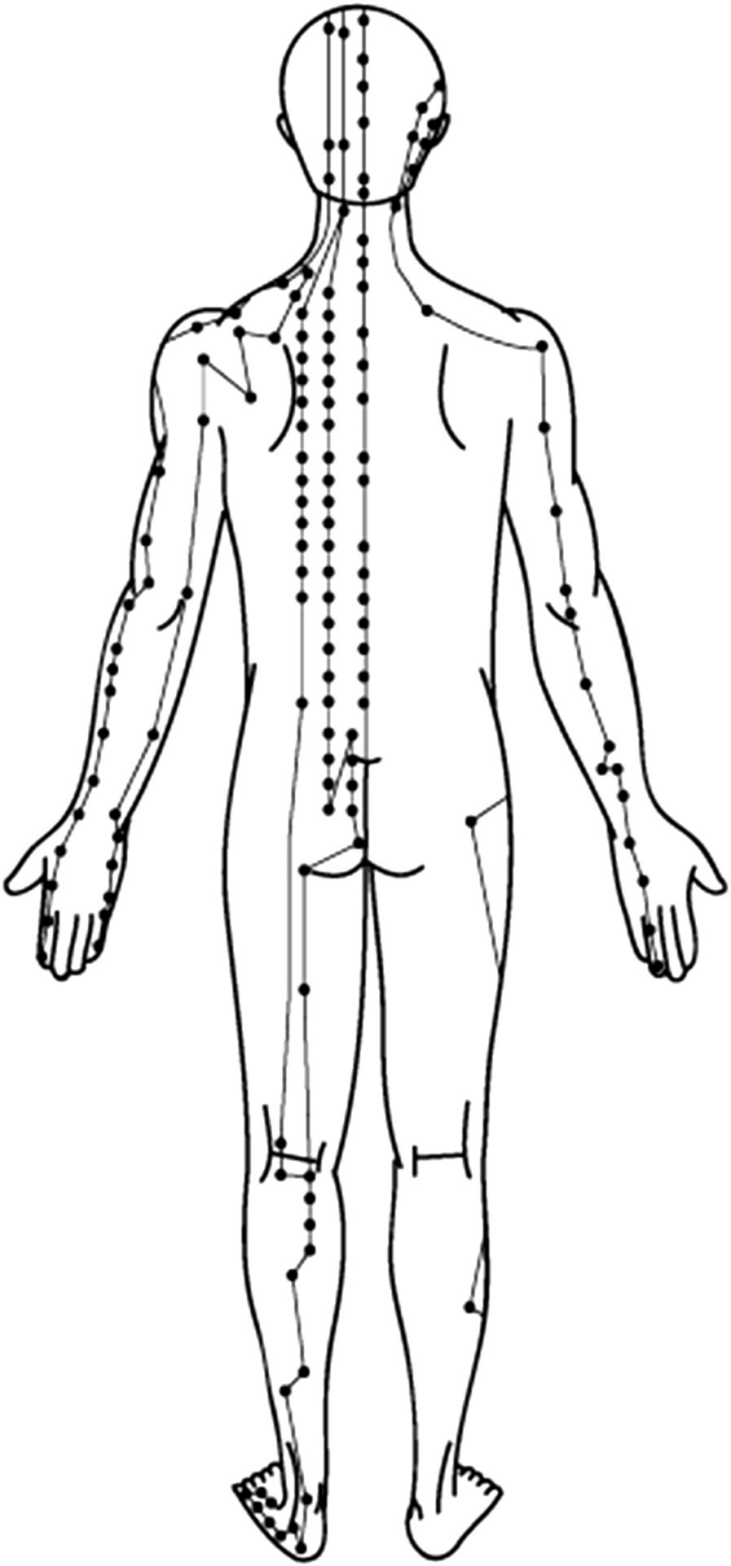
Meridian: example of the meridian points.

**Figure 2:**
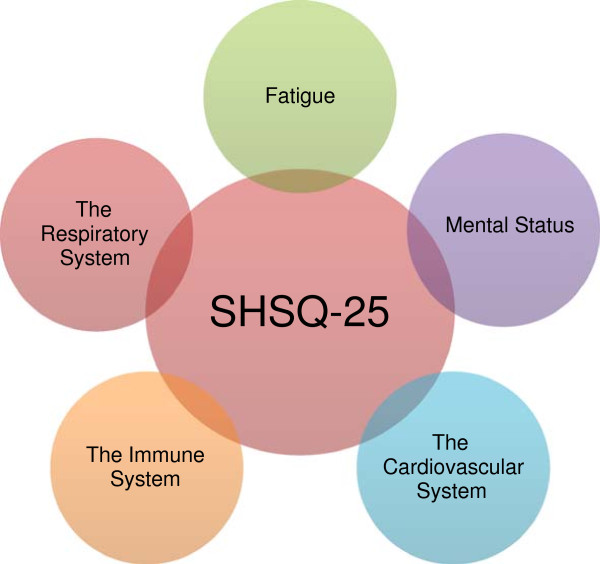
Suboptimal health: five domains.

China is a country with 5,000 years of civilization, and traditional Chinese medicine (TCM) is one of the prestigious medical heritages in the world, with over 2 millennia of clinical practices [7,8]. The constituents in TCM preparations are influenced by three principal factors: heredity (genetic composition), ontogeny (stage of development) and environment (climate, associated flora, soil and method of cultivation). The concept of *Jing* (meridian), one of the foundational principles of TCM, plays a central role in the TCM theoretical frame (Figure 1). Presumably, *Jing* has referred to the genetic information as well as its plasticity, as *Jing* is thought to be ‘the substance essential for development, growth and maturation’ and ‘conception is made possible by the power of *Jing*, growth to maturity is the blossoming of *Jing*, and the decline into old age reflects the weakening of the *Jing*’ [8]. But unfortunately, some of the TCM conceptions have not been recognized internationally due to the lack of systemic evidenced supports [8]. Suboptimal health status (SHS) is such an example. Since the ancient time, TCM has been identifying a physical status between health and disease which we coined as SHS [2,9,10]. SHS is a physical state between health and disease and is characterized (1) by the perception of health complaints, general weakness and low energy within a period of 3 months; and (2) as a subclinical, reversible stage of chronic disease [2,9,10] (Figure 2).

**Figure 3:**
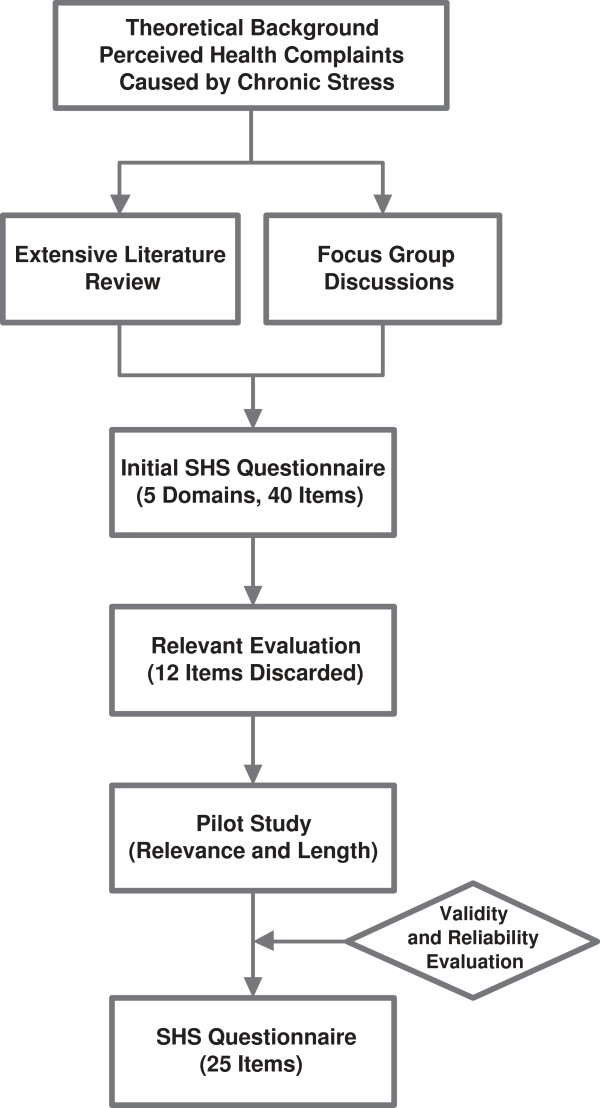
Workflow of SHSQ-25 development.

The existence of a reliable and valid instrument to assess SHS is essential. We, therefore, developed and validated a comprehensive suboptimal health status questionnaire-25 (SHSQ-25) to assess SHS among urban Chinese [9,10]. The SHSQ-25 accounts for the multidimensionality of SHS by encompassing the following domains: (1) fatigue, (2) the cardiovascular system, (3) the digestive tract, (4) the immune system and (5) mental status (Figures 2 and 3). The SHSQ-25 is short and easy to complete and, therefore, an instrument suitable for use in both large-scale studies of the general population and routine health survey [2,9,10].

The examples on the validation of the SHSQ-25 instrument and the application of the SHSQ-25 on the association study of SHS and cardiovascular risk factors in urban Chinese workers were reported in our recent publications [9,10]. Herein is a brief summary of SHS study on the urban Chinese workers in Beijing.

## Methods

### SHSQ-25 development

**Figure 4:**
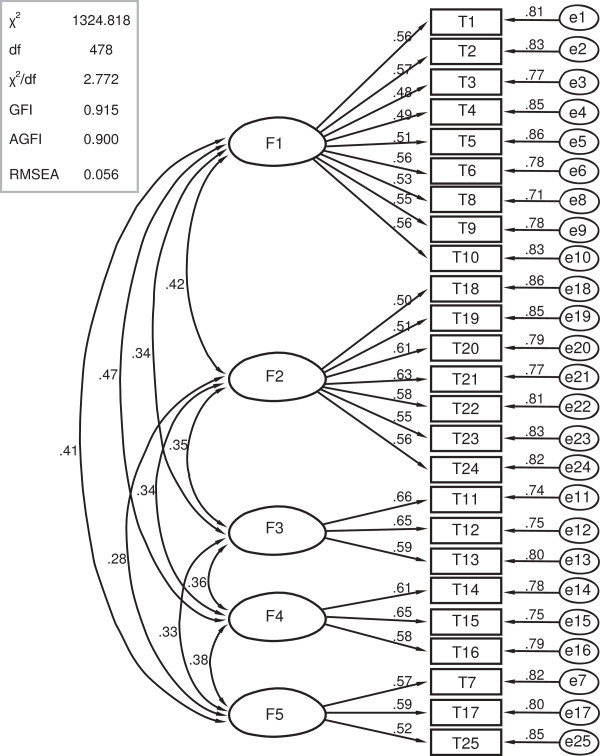
Confirmatory analysis of the five domains and 25 elements of the SHSQ-25.

The development and validation of SHSQ-25 were described in the previous publications [9,10]. Figure 4 shows the confirmatory analysis of the five domains with a total of 25 elements in the instrument of SHSQ-25 (Figure 4), and Figure 3 gives a schematic presentation overviewing the detailed workflow of SHSQ-25 (Figure 3).

### Study design and participant recruitment

A cross-sectional study was conducted among workers in urban Beijing. A random third of the 64 Beijing-based companies whose workers took annual physical examination for at least two consecutive years at the physical examination center affiliated to Capital Medical University, Beijing, China were selected for cluster sampling (Table 1). The Capital Medical University research ethical committee approved the study, and written informed consent was obtained from all participants. The participants had to meet the following inclusion criteria: (1) no history of somatic or psychiatric abnormalities, as confirmed by their medical records; (2) age from 20 to 60 years; and (3) no history of medication consumption in the previous 2 weeks. All participants attended a standardized examination protocol in the physical examination center, including medical history, physical examination, blood hematology and biochemistry analysis, rest electrocardiography and abdominal ultrasonography. We excluded individuals who met the diagnostic criteria of specific diseases concerning the cardiovascular system, the respiratory system, the genitourinary system, the digestive system, the hematic system, and diabetes. Of the 4,881 workers, 3,405 people met the criteria and were further investigated.

**Table 1:** **Characteristics of study sample**[10]

**Variables**	**SHS score ≥ 44 (*n *** **(%))**	**SHS score ≤ 44 (*n *** **(%))**	***χ *** **2 value**	***P *** **value**
Sex				
Female	806 (52.10)	660 (44.84)	15.933	<0.001
Male	741 (47.90)	812 (55.16)		
Age (years)				
20–30	167 (10.80)	376 (25.54)	128.978	<0.001
31–40	606 (39.17)	575 (39.06)		
41–50	559 (36.13)	380 (25.82)		
51–60	215 (13.90)	141 (9.58)		
Education level				
Compulsory school (through to grade 9)	72 (4.65)	171 (11.62)	185.420	<0.001
High school graduate	213 (13.77)	432 (29.35)		
University/college degree	1,262 (81.58)	869 (59.04)		
Occupation				
White-collar worker	1,431 (92.50)	986 (66.98)	307.665	<0.001
Blue-collar worker	116 (7.50)	486 (33.02)		
Monthly income (RMB)				
<2,000	324 (20.94)	316 (21.47)	3.852	0.146
2,001–5,000	1,012 (65.42)	990 (67.26)		
≥5,000	211 (13.64)	166 (11.28)		
Marital status				
Single or divorced	196 (12.67)	168 (11.41)	1.123	0.289
Married	1,351 (87.33)	1,304 (88.59)		
Current smoking				
Yes	476 (30.77)	187 (12.70)	143.638	<0.001
No	1,071 (69.23)	1,285 (87.30)		
Alcohol use				
Every day	72 (4.65)	54 (3.67)	2.309	0.511
3–4/week	351 (22.69)	348 (23.64)		
1–2/week	988 (63.87)	948 (64.40)		
Never	136 (8.79)	122 (8.29)		
Physical activity (hour)				
≥5	44 (2.84)	47 (3.19)	31.772	<0.001
3–4	341 (22.04)	437 (29.69)		
1–2	641 (41.44)	486 (33.02)		
<1	521 (33.68)	502 (34.10)		

### SHSQ-25 survey

The SHSQ-25 self-reported questionnaire was used to assess the respondents' socio-demographics and SHS. Median was used as a cut point in grouping into high versus low of the two dimensions of SHS (Additional file 1). To assure comparability of the findings, all participants were examined by the physicians who were specially trained for the study. SHSQ-25 includes five domains of 25 items [6]. Each subject was asked to rate a specific statement on a five-point Likert-type scale, based on how often they suffered various specific complaints in the preceding 3 months: (1) never or almost never, (2) occasionally, (3) often, (4) very often and (5) always. The raw scores of 1 to 5 on the questionnaire were re-coded as 0 to 4. SHS scores were calculated for each respondent by summing the ratings for the 25 items.

### Laboratory-based tests

Overnight fasting blood specimens were obtained for the measurement of plasma glucose, serum lipids and cortisol. Plasma glucose was measured using a modified hexokinase enzymatic method (Hitachi automatic clinical analyser, model 7060, Chiyoda, Tokyo, Japan). Concentrations of total cholesterol, high-density lipoprotein (HDL) cholesterol and triglycerides (TG) were assessed enzymatically with commercially available reagents. Lipid measurements were standardized according to the criteria of the Centers for Disease Control and the Prevention - National Heart, Lung, and Blood Institute Lipid Standardization Program. Low-density lipoprotein (LDL) cholesterol was computed by the Friedewald formula using the equation [LDL = total cholesterol and (HDL + TG / 5)] [8]. Fasting cortisol was analyzed by γ radioimmunoassay (RIA) counter (GC-911). The intra-assay and inter-assay coefficient of variations (CVs) were <5.5 % and <7.5 %, respectively. The reference value was 50–280 ng/ml.

### Quality controls

Quality controls were applied throughout all these assays. Body weight and height were measured twice during the interview. Weight was measured in light indoor clothing without shoes on electronic scales placed on a firm, level surface to the nearest 0.1 kg. Height was measured without shoes with a wall-mounted stadiometer to the nearest 0.1 cm. Body mass index (BMI) was calculated as body weight (in kilogram) divided by height (in metre) squared. Blood pressures were measured twice on the right arm by well-trained nurses using a standard mercury sphygmomanometer with the subjects resting at least 5 min in a sitting position. Data on socio-demographic information and health-related behaviors were collected during the interviews and used to control for potential confounding in analyses. Demographic variables included age, education, occupation, average monthly income and marital status. The health-related behaviors included smoking status, alcohol use and physical activity. Smoking status was dichotomized as current smoker (≥1 filter per day) and non-smoker. Physical activity was assessed by asking to list the average hours of physical activity for each day of the week prior to the questionnaire.

### Data analysis

Data were reported as mean ± SD for continuous variables and frequencies for categorical variables. Univariate and multivariate analyses were performed to estimate the relations of SHS to risk factors for cardiovascular disease. A two-level model was used in multivariate analysis to account for the nested nature of the data, with ‘sampling company’ and ‘study participant’ as the first and second levels, respectively. Descriptive statistics and univariate analysis were carried out using SAS version 8.2. For multilevel analysis, we used MLwin software (version 2.02, 2005). The *P* values for the fixed part of the model were obtained by calculating the Chi square statistics for joint contrasts provided by the MLwin software package. Value of *P* < 0.05 was considered statistically significant.

## Results

### SHS survey

The response rate was high (88.7%), which therefore limited the potential selection bias. Among the 4,881 participants, our analysis was restricted to 3,019 individuals who had completed both the questionnaire and laboratory results. Their mean age was 40.6 years (SD 13.4) and 48.6% were women. The participants were divided into two groups by the median SHS score of 44: high SHS score group (SHS score ≥44) and low SHS score group (SHS <44). The mean SHS score among the SHS group was 55.73 ± 9.58 and 35.02 ± 6.51 among the control group, respectively (Table 2). Gender and age distribution were also different between the two groups (*P* < 0.001). Since over half of the individuals aged over 50 years were excluded because of the results of their general medical examination, the number of this age group was significantly lower than the younger age groups. SHS was correlated with current smoking and physical inactivity (*P* < 0.001); whereas monthly income, marital status and alcohol use were not different between groups. Overall, the participants who gave higher SHS score also had a higher risk of cardiovascular disease than those with lower scores. Compared to the low-score group, systolic and diastolic blood pressure, plasma glucose, total cholesterol, TG levels and BMI were significantly higher among the high-score group (*P* < 0.001).

**Table 2:** **Comparison of the cardiovascular risk factors between high and low SHS score group**[10]

	**SHS score high Mean + SD**	**SHS score low Mean + SD**	***t *** **value**	***P *** **value**
SBP (mmHg)	119.43 ± 13.27	115.31 ± 13.19	8.573	<0.001
DBP (mmHg)	77.57 ± 7.38	75.38 ± 7.89	7.880	<0.001
GLU (mmol/L)	5.23 ± 0.57	5.17 ± 0.55	2.941	<0.001
TCH (mmol/L)	4.48 ± 0.76	4.32 ± 0.78	5.708	<0.001
TG (mmol/L)	1.17 ± 0.58	1.08 ± 0.46	4.709	<0.001
HDLC (mmol/L)	1.32 ± 0.32	1.36 ± 0.36	-3.230	<0.001
LDLC (mmol/L)	2.82 ± 0.70	2.78 ± 0.71	1.558	0.119
COR (ng/ml)	204.31 ± 40.06	161.33 ± 27.83	34.076	<0.001
BMI (kg/m^2^)	23.24 ± 3.76	22.01 ± 3.52	9.268	<0.001

*SHS* suboptimal health status, *SBP* systolic blood pressure, *DBP* diastolic blood pressure, *GLU* plasma glucose, *TCH* total cholesterol, *TG* triglyceride, *HDLC* high-density lipoprotein cholesterol, *LDLC* low-density lipoprotein cholesterol, *COR* serum cortisol.

### Laboratory test

HDL cholesterol levels were higher in low-score group than in high-score group (*P* > 0.05). Serum cortisol level was much higher among the high-score group than that among the low-score group (204.31 vs. 161.33 ng/ml, *P* < 0.001). The ranges of cortisol in high-score and low-score group were 122.64–324.17 and 107.12–221.59 ng/ml, respectively. A significant linear correlation between SHS score and serum cortisol was evident (*r* = 0.381, *P* < 0.001). After adjusting for age, education background, occupation, smoking and physical activity, the diastolic blood pressure, plasma glucose, total cholesterol and serum cortisol were found to significantly predict SHS score (*P* < 0.05) among men. HDL cholesterol levels were negatively and significantly associated with SHS score (*P* < 0.05). In the fully adjusted multilevel analysis, no significant association was observed among TG, LDL cholesterol, BMI and SHS score (*P* > 0.05). As the major risk factors for most chronic diseases, smoking and lack of physical activity were also significantly associated with SHS score among men (*P* < 0.05) (Table 3). The results of parameter estimates and standard errors from the two-level model among women were similar to those among men, with the exception of plasma glucose and HDL cholesterol (Table 4).

**Table 3:** **Multilevel estimates for SHS score in relation to cardiovascular risk factors among male participants**[10]

	**Estimate**	**SE**	***P *** **value**
Systolic blood pressure	0.601	0.211	0.004
Diastolic blood pressure	0.486	0.230	0.035
Plasma glucose	0.636	0.302	0.035
Total cholesterol	1.003	0.333	0.003
Triglyceride	0.477	0.293	0.104
HDL cholesterol	-0.986	0.400	0.014
LDL cholesterol	0.160	0.116	0.168
Serum cortisol	0.231	0.004	<0.001
Body mass index	0.180	0.214	0.400
Level 2 intercept variance (person)		6.903 (1.369)	
Level 2 intercept variance (company)		3.418 (1.192)	

*HDL* high-density lipoprotein, *LDL* low-density lipoprotein.

**Table 4:** **Multilevel estimates for SHS score in relation to cardiovascular risk factors among female participants**[10]

	**Estimate**	**SE**	***P *** **value**
Systolic blood pressure	0.388	0.181	0.032
Diastolic blood pressure	0.751	0.280	0.007
Plasma glucose	0.151	0.116	0.193
Total cholesterol	1.353	0.423	0.001
Triglyceride	1.245	0.407	0.002
HDL cholesterol	-1.516	0.669	0.024
LDL cholesterol	0.420	0.365	0.250
Serum cortisol	0.225	0.005	<0.001
Body mass index	0.250	0.197	0.205
Level 2 intercept variance (person)		4.152 (1.530)	
Level 2 intercept variance (company)		2.414 (1.116)	

*HDL* high-density lipoprotein, *LDL* low-density lipoprotein.

### Key findings

Based on our study, we present the following key findings:

1. The prevalence of SHS in urban Chinese is around 14.3%. SHS is more prevalent in women than in men and more in white-collar workers than in blue-collar workers.

2. We found a correlation between SHS and systolic blood pressure, diastolic blood pressure, plasma glucose, total cholesterol and HDL cholesterol among men, and a correlation between SHS and systolic blood pressure, diastolic blood pressure, total cholesterol, TG and HDL cholesterol among women.

3. In addition, the prevalence of SHS increases with age. This trend is consistent with the prevalence of metabolic syndrome and cardiovascular disease in urban China.

4. The similarity could partially be accounted by sharing common risk factors. Significantly higher level of serum cortisol among high SHS score group compared to low-score group and significant linear correlation between SHS score and serum cortisol strengthen the evidence that stress is an important related factor for SHS.

5. The SHSQ-25 is a self-rated questionnaire of perceived health complaints, which can be used as a new instrument for predictive, preventive and personalized medicine (PPPM).

## Expert recommendations

Currently, there are no tests that can confirm or exclude SHS. Rather, it is a disease of exclusion made after common physical and mental causes of the symptoms have been excluded, and predisposing factors, triggering events and maintenance factors have been identified [9-11]. The etiology of SHS remains unclear, with both psychiatric/psychological causes and physical causes such as a viral illness and metabolic disequilibrium being considered. The newly created SHSQ-25 is an instrument that attempts to measure the SHS qualitatively, which is short and easy to complete, and therefore an instrument suitable for use in both large-scale studies of the general population and routine health survey.

With the rapid economic progress across China, people have been becoming more exposed to stressful situations such as excessive workload, competition and perceived loneliness [9]. Continuous psychosocial stress seems to be a part of the everyday life in China, especially among white-collar workers [12-14]. Endocrine measures of stress and self-rated health (SRH) were also proved in a longitudinal study. Poorer SRH at each time point was associated with higher levels of serum cortisol and prolactin [15]. SRH may capture subclinical or undiagnosed disease [16]. In addition, the measures of perceived health modified the effects of biomedical risk factors, both in the prediction of myocardial infarction and stroke [13,14]. A possible pathway for the observed effects is that SRH reflects the presence or absence of psychosocial risk factors or resources [17]. Psychological stress can affect health not only directly through neuro-endocrine responses but also indirectly through changes in health behaviors [18]. Current smoking was significantly more common in individuals giving higher SHS score. They also reported significantly less physical activity.

In China, non-communicable chronic diseases (NCD) accounts for about 80% of deaths and 70% of disability-adjusted life years [19,20]. As a developing country with a population of 1.3 billion, it is imperative that an economical and valid instrument is developed for screening major chronic diseases. In many developed counties, much attention has been paid on perceived poor health, such as chronic fatigue syndrome ‘CFS’, somatization and unexplained medical syndrome ‘UMS’ in community and primary care system [21,22]. Somatic symptoms are one of the main reasons for patients seeking health care [23,24]. SHS cannot be fully understood from the conventional disease-oriented biomedical point-of-view. Instead, it requires a holistic bio-psychosocial perspective in which complaints are viewed as the result of complex interactions of physiology, psychology and social environment. Primary care providers must be able to detect and manage SHS to fight the delayed intervention, untargeted medication, overdosed and poisoned patients, and ineffective treatments [25]. The SHSQ-25 is a valid instrument for such purpose from the perspective of PPPM. Effective intervention on SHS may be a cost-effective way for preventing chronic diseases. In addition, laboratory-based screening and development of disease-related biomarkers (genomics, proteomics, glycomics and metabolomics) is also the key promise for the early diagnosis, prevention and management in the practice of PPPM [26-28], and for improving the ecology of medical care globally [25,28-31].

For both developing and developed countries, the integrative concept of PPPM would enable clinicians and public health workers to predict an individual's predisposition in order to provide targeted preventive measures before the actual onset of the disease. Using the innovative PPPM tool, such as SHSQ25, biomarkers (medical imaging or pathology-specific molecular patterns, sub/cellular imaging and omics), pharmacogenetics, disease and patient modeling, individual patient profiles and a combination of Western and traditional Chinese medicine, the expected outcomes are conducive to more effective population screening, prevention measures early in life, identification of persons who are at risk, stratification of patients for optimal therapy planning and prediction and reduction of adverse drug-drug or drug-disease interactions. The integrative approach by PPPM is thus considered as the optimal platform of translational medicine [2,25,31].

### Outlook

With the support of the rapid progress of biotechniques and the availability of mega-health databases such as Human Genome Project, health professionals are in a good position to address the topics genetics, environment and behaviors and to motivate the introduction of PPPM into daily medical services (Table 5).

**Table 5:** Topics which motivate the introduction of PPPM into daily medical services

Gene	vs.	Environment
Nature	vs.	Nurture
Genomics	vs.	Genetics (epigenetics)
Rare disease	vs.	Common diseases
Infectious diseases	vs.	Non-communicable chronic diseases
Public health	vs.	Individualized medicine
Western medicine	vs.	Traditional medicine
Predictive, preventive	vs.	Treatments
Nutrition	vs.	Exercise
Males	vs.	Females
Children	vs.	Adults
City	vs.	Rural
Rich	vs.	Poor
Obesity	vs.	Malnutrition
Health	vs.	Disability
Developed countries	vs.	Developing countries
Migrants	vs.	Residences

From the perspective of Western medicine, the innovative tools, pointing to an awareness of the totality of human health, have arisen as a direct outcome of the Western medicine urge to penetrate phenotypes and to unravel the transcendent truth behind them. Western medicine has been nourished by the constant tension between the unknown and known, and imperfect and perfect [7,8]. In the initiative of PPPM, the European Association for Predictive, Preventive and Personalised Medicine issued a timely white paper in 2013, summarizing the priorities of PPPM: ‘Healthcare in overview across the globe’, ‘PPPM in diabetes mellitus’; ‘Multimodal approaches in female healthcare’, ‘PPPM in cardiovascular diseases’, ‘PPPM in cancer’, ‘PPPM in neurodegenerative disease’, ‘Targeted prevention in nutrition, behaviour and physical activity’, ‘Anti ageing’, ‘Priorities of PPPM in dentistry’, ‘Patient specific modelling’, ‘Biomarker discovers, validation, standardization and practical application in medical practice’ and ‘Biobank’ [31]. To achieve the goals, seven work groups (WG1-PPPM in Pre/Diabetes Care, WG2-PPPM in Cardiovascular Disease, WG3-PPPM in Cancer, WG4-PPPM in Neurological, Neuropsychiatric and Neurodegenerative Diseases, WG5-PPPM in Dental and Oral Pathologies, WG6-PPPM in Infectious Diseases and WG7-PPPM in Rare Diseases) were set accordingly in year 2013 to translate PPPM into practices; and the work group consists of 34 COST-, COST-near neighbour and COST international partner countries according to the European Cooperation in Science and Technology (COST) Trans-domain Proposal (TDP) guidelines [32].

In TCM on the other hand, the Chinese doctors direct their attention to the complete individual, including psychological aspects that western physicians often see as unrelated to a specific health and disease issue. All relevant information, including the symptoms as well as the patient's other general characteristics, is gathered and woven together until it forms what TCM called a ‘pattern of disharmony’ [7,8]. This pattern of disharmony describes a situation of ‘distress’ or ‘imbalance’ in a patient's body. The oriental diagnostic technique was described as ‘It does not turn up a specific disease entity or precise cause, but renders an almost poetic, yet workable, description of a whole person. The therapy then attempts to bring the configuration into balance, to restore harmony to the individual’ [7]. Taking the merits of both disciplines, SHSQ-25 is the tool developed based on the combination of both TCM and Western medicine principles. An ongoing longitudinal SHS cohort survey (China Sub-optimal Health Cohort Study, COACS) consisting of 50,000 participants will provide a powerful health trial to use SHSQ-25 for its application to PPPM through patient stratification and therapy monitoring using innovative technologies of predictive diagnostics and prognosis: an effort of paradigm shift from reactive to predictive medicine.

## Conclusion

The questionnaire SHSQ-25 congregated into a score (five health domains with 25 components) which could significantly distinguish among several abnormal conditions and could be used as an effective instrument for PPPM practice.

## Competing interests

The authors declare that they have no competing interests.

## Authors’ contributions

WW designed the survey, and drafted, completed and revised the manuscript. AR edited the manuscript, tables and figures. YXY carried out survey and analyzed the data. All authors read and approved the final manuscript.

## Supplementary Material

Additional file 1 The SHSQ-25 questionnaire. Click here for file
