# Erratum: Traditional Chinese medicine and new concepts of predictive, preventive and personalised medicine in diagnosis and treatment of sub-optimal health

**DOI:** 10.1186/1878-5085-5-12

**Published:** 2014-08-26

**Authors:** Wei Wang, Alyce Russell, Yuxiang Yan

**Affiliations:** 1 School of Medical Sciences, Edith Cowan University, Perth, Western Australia WA6027, Australia; 2 Municipal Key Laboratory of Clinical Epidemiology, School of Public Health, Capital Medical University, Beijing 100069, China

## Erratum

Following publication of our article [1] we noticed that we included the incorrect version of figure two (Figure 1 here) representing the 5 domains of suboptimal health. We have provided the correct figure here.

**Figure 1:**
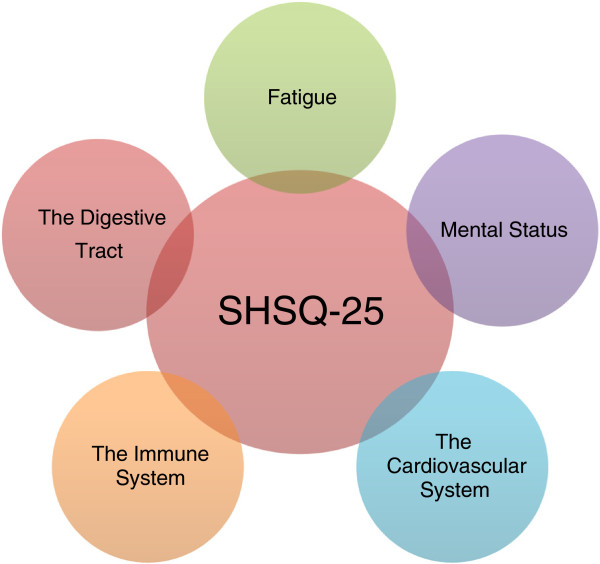
Suboptimal health: five domains.

We would also like to clarify that the data presented in tables 1–5 in the article [1] have previously been published in our earlier article [2]. The recent article uses the original data but provides a new interpretation based on the innovative paradigm of predictive, preventive and personalized medicine. The authors apologise for not clearly stating this in our recent article and for any confusion caused.
