# Supplementary material for: Traditional Chinese medicine and new concepts of predictive, preventive and personalized medicine in diagnosis and treatment of suboptimal health
Source: EPMA J. 2014 Feb 13;5(1):4. doi: 10.1186/1878-5085-5-4 (PMC3926271; doi:10.1186/1878-5085-5-4)
Supplement: Additional file 1 — The SHSQ-25 questionnaire. [file 1878-5085-5-4-S1.pdf]

Suboptimal health

Welcome to the Suboptimal Health Survey

THE PURPOSE

The rapid environmental changes that accompany urbanisation are increasing the prevalence of the major risk factors for chronic diseases, including work stress, physical inactivity, unhealthy diet, and tobacco use. The purpose of this project is to gather information about health events during the last 3 months amongst Australian population to assess their suboptimal health status.

ABOUT PARTICIPATION

You have been invited to the online survey, which will take approximately 10 minutes to complete. Your participation in this project is entirely voluntary. If you do participate, you can withdraw from the project at any time without comment or penalty. Any identifiable information already obtained from you will be destroyed.

Participation will involve completing a Suboptimal Health Status questionnaire that includes 25 questions. Each question will have 5 responses and you have to choose the one that you consider the most appropriate. The questionnaire cover the five domains of fatigue, the cardiovascular system, the digestive system, the immune system, and the mental status. It is short and easy to complete, and therefore suitable for use in studies of the general population.

EXPECTED RISKS AND BENEFITS

There are no risks beyond normal day-to-day living associated with your participation in this project.

It is not expected that this project will directly benefit you. However, it will assist improving knowledge in the field of this growing public health concern in Australia. You will not be recompensed for your participation.

ABOUT PRIVACY AND CONFIDENTIALITY

All comments and responses will be treated confidentially and will be made anonymous for analysis. The names of individual persons are not required in any of the responses. Please note that non-identifiable data collected in this project may be used as comparative data in future projects.

ABOUT THE SURVEY AND DATABASE MANAGEMENT

This project adheres to ECU's policies relating to data collection from human subjects, and has been approved by the ECU Human Research Ethics Committee. User data and records will be held in a secure database, accessible only to the researcher and project leader. Stored data will be held securely for a period of at least 5 years. In keeping with Australian Federal Privacy Legislation, the identity of individual's involved in the user-group will be held in the strictest of confidence, including email addresses and/or any contact details. Results reported in any aggregated 'findings' will not be associated with the identity of any one individual. Analysed and cross-analysed data (i.e., results and findings) may be used in associated ECU research projects outside of the current project, however raw user data, in particular that data which may identify individual participants or sensitive research career data will be used for the expressed purpose of the current research project only.

Suboptimal health

FOR QUESTIONS OR CONCERNS

For questions or concerns relating to this study please contact Professor Wei Wang at 6304 3717 or wei.wang@ecu.edu.au

An independent contact person is also available should a user feel their concern/enquiry has not been dealt with adequately. In this case, please contact Research Ethics Officer at 6304 2170 or research.ethics@ecu.edu.au

CONSENT TO PARTICIPATE

I have read and I freely agree to participate in this project.
I understand that consent is implied by electing to commence the online survey.

Characteristics of questionnaire respondents.

*** 1. Gender**

Male	Female
○	○

*** 2. Age (years)**

18 - 30	31 - 40	41 - 50	51 - 60
○	○	○	○

*** 3. Highest level of education.**

Compulsory education-through grade 9	High school graduation	University / college degree
○	○	○

*** 4. Occupation**

White-collar worker	Blue-collar worker	College student	Other
○	○	○	○

SHSQ-25 - Thinking about your health in the past 3 months

*** 5. In the past 3 months how often were you exhausted without greatly increasing your physical activity?**

never or almost never	occasionally	often	very often	always
○	○	○	○	○

*** 6. In the past 3 months how often did you have fatigue which could not be substantially alleviated by rest?**

never or almost never	occasionally	often	very often	always
○	○	○	○	○

*** 7. In the past 3 months how often were you lethargic when working?**

never or almost never	occasionally	often	very often	always
○	○	○	○	○

Suboptimal health

∗8. In the past 3 months how often did you suffer from headaches?

never or almost never	occasionally	often	very often	always
○	○	○	○	○

∗9. In the past 3 months how often did you suffer from dizziness?

never or almost never	occasionally	often	very often	always
○	○	○	○	○

∗10. In the past 3 months how often did your eyes ache or feel tired?

never or almost never	occasionally	often	very often	always
○	○	○	○	○

∗11. In the past 3 months how often did you suffer from a sore throat?

never or almost never	occasionally	often	very often	always
○	○	○	○	○

∗12. In the past 3 months how often did your muscles or joints feel stiff?

never or almost never	occasionally	often	very often	always
○	○	○	○	○

∗13. In the past 3 months how often did you have pain in your shoulders / neck / waist?

never or almost never	occasionally	often	very often	always
○	○	○	○	○

∗14. In the past 3 months how often did you have a heavy feeling in your legs when walking?

never or almost never	occasionally	often	very often	always
○	○	○	○	○

∗15. In the past 3 months how often did you feel out of breath while resting?

never or almost never	occasionally	often	very often	always
○	○	○	○	○

∗16. In the past 3 months how often did you suffer from chest congestion?

never or almost never	occasionally	often	very often	always
○	○	○	○	○

∗17. In the past 3 months how often were you bothered by heart palpitations?

never or almost never	occasionally	often	very often	always
○	○	○	○	○

∗18. In the past 3 months how often was your appetite poor?

never or almost never	occasionally	often	very often	always
○	○	○	○	○

∗19. In the past 3 months how often did you suffer from heartburn?

never or almost never	occasionally	often	very often	always
○	○	◉	○	○

Suboptimal health

***20. In the past 3 months how often did you suffer from nausea?**

never or almost never	occasionally	often	very often	always
◯	◯	◯	◯	◯

***21. In the past 3 months how often did you have difficulty tolerating the cold?**

never or almost never	occasionally	often	very often	always
◯	◯	◯	◯	◯

***22. In the past 3 months how often did you have difficulty falling asleep?**

never or almost never	occasionally	often	very often	always
◯	◯	◯	◯	◯

***23. In the past 3 months how often were you troubled by waking up during night?**

never or almost never	occasionally	often	very often	always
◯	◯	◯	◯	◯

***24. In the past 3 months how often did you have trouble with your short-term memory?**

never or almost never	occasionally	often	very often	always
◯	◯	◯	◯	◯

***25. In the past 3 months how often did you have difficulty responding quickly?**

never or almost never	occasionally	often	very often	always
◯	◯	◯	◯	◯

***26. In the past 3 months how often did you have difficulty concentrating?**

never or almost never	occasionally	often	very often	always
◯	◯	◯	◯	◯

***27. In the past 3 months how often were you distracted for no reason?**

never or almost never	occasionally	often	very often	always
◯	◯	◯	◯	◯

***28. In the past 3 months how often did you feel nervous or jittery?**

never or almost never	occasionally	often	very often	always
◯	◯	◯	◯	◯

***29. In the past 3 months how often did you catch a cold?**

never or almost never	occasionally	often	very often	always
◯	◯	◯	◯	◯

Thank You for your time to complete this survey.

DISCLAIMER

If after participating in this survey, you have any concerns about your physical or mental health, please consult your doctor without delay. If you would like contact details of any counseling services or support groups, please contact the researcher **Professor Wei Wang at 6304 3717 or wei.wang@ecu.edu.au**.
